# The Role of Body Shame and Age on Appearance-Based Exercise and Positive Body Image in Women from Poland: Preliminary Results of a Cluster Analysis

**DOI:** 10.3390/ijerph192315741

**Published:** 2022-11-26

**Authors:** Kamila Czepczor-Bernat

**Affiliations:** Department of Pediatrics, Pediatric Obesity and Metabolic Bone Diseases, Faculty of Medical Sciences in Katowice, Medical University of Silesia, 40-055 Katowice, Poland; k.czepczor.bernat@gmail.com

**Keywords:** body shame, age, appearance-based exercise, positive body image, women

## Abstract

The aim of this study has been to analyse whether body shame and age may play a role in appearance-based exercise and positive body image in Women from Poland. It was assumed that women with high body shame and at the stage of young adulthood have significantly greater levels of appearance-based exercise and lower positive body image than those with a low level of body shame and at the stage of middle adulthood. The final sample included 234 Polish women (age: *M* = 31.58, *SD* = 13.93; body mass index: *M* = 23.45, *SD* = 4.72). Participants completed: the Objectified Body Consciousness Scale (OBCS), the Exercise Appearance Motivations Scale (EAMS), the Body Appreciation Scale-2 (BAS-2) and a socio-demographic survey. The cluster analysis technique indicated four distinct clusters: (a) Cluster 1 (*N* = 83): high body shame and young adulthood; (b) Cluster 2 (*N* = 29): high body shame and middle adulthood; (c) Cluster 3 (*N* = 88): low body shame and young adulthood; (d) Cluster 4 (*N* = 34): low body shame and middle adulthood. The outcomes partially support the hypothesis, as higher levels of almost all subscales related to appearance-based exercise (EAMS: “muscularity”, “societal pressures”, “shape/weight concerns”, “avoidance/shame”) and lower positive body image (BAS-2) were observed in women with high body shame and at the stage of young adulthood compared with women with a low level of body shame and at the stage of middle adulthood. These results indicate that both body shame and age may contribute to the intensity of appearance-based exercise and positive body image deterioration. Clarity on this issue is essential to ensure that an appropriate preventive activity and interventions are made which will be able to take into account the specific sociocultural context in Poland.

## 1. Introduction

Since the 1990s the objectification theory has been the basis for multiple studies taking into account the basic components of this structure, such as body shame, body surveillance and control beliefs [1,2,3]. Many of these analyses focused on the subject of experiences related to the human body, including body image in particular, e.g., [4,5,6]. Body image is defined as a subjective mental representation of one’s own body and appearance [7,8]. It is a multidimensional concept that has to do with the thoughts and beliefs, emotions, and behaviours related to the body [7,8]. Body image is a social construction and is a factor that affects females as well as males [8,9,10,11,12]. What is important, however, is how the social perception of women’s bodies affects the formation and maintenance of body image in this group [8,9,10,11,12]. This is mentioned, among others, by the Tripartite Influence Model of Body Image [8,10,11,12]. It assumes that the media, peers and family are the main source of information about what kind of body is desirable and considered attractive (standards of appearance in society). These three sociocultural sources of influence may strengthen the tendency to compare one’s body with the “ideal”, which may often be impossible to achieve for most people and/or have a strong negative impact on physical and mental health. This feminine beauty ideal is internalized over time and may directly lead to strong body dissatisfaction (and related body shame) and indirectly to the appearance of symptoms of eating disorders, symptoms of depression and deterioration of overall mental functioning [8,10,11,12]. Importantly in this context, previous research shows that women may be at increased risk for developing the difficulties described above, as they seem to be more susceptible to body shaming compared to men [9], which is consistent with reports indicating a clearly stronger objectification of the female body in Western countries (related to, inter alia, treating the body as an object that should meet certain standards, including being a source of sexual pleasure for others (sexualization)) and more frequent identification of a woman’s worth through the prism of her appearance than in the group of men [13,14].

When discussing body image, it should be mentioned that it can be positive or negative [15,16]. When analysing the literature, it is easy to notice that there are many studies showing that negative body image is associated with body shame, e.g., [6]. However, little is known about how shame towards the body can affect the construct of a positive body image. It seems important to conduct research in this area due to the fact that, as the world experts in the field of body image examination indicate, a positive body image is a structure separate from a negative body image [16]. This means that a positive body image is not the opposite side of the continuum for positive body image (a positive body image is therefore not just an expression of a low level of negative body image) [16]. Positive image may be understood as love and respect for your own body expressed, inter alia, through [16,17]: (a) accepting your own body (even if it does not conform to idealized standards of beauty), (b) appreciating the uniqueness of the body and functions it can perform in our lives, (c) feeling comfortable and being happy in relation to your body, (d) the ability and tendency to appreciate the values of one’s body, and not to focus too much on its flaws. It is therefore worth verifying whether the direction of the relationship between body shame and positive body image will be consistent with the outcomes from research on negative body image—i.e., whether a high level of shame will favour a low level of positive body image, e.g., [6,18,19].

Feeling negative emotions towards the body may predispose a person to take various actions that will allow change to a selected unaccepted aspect of corporeality (e.g., weight, figure, musculature) or general attitude to their own body [7,8,20,21]. However, these activities are not always pro-health because it is not uncommon for people to forget about health while practising physical activity, and not adjust the type and intensity of exercise to their abilities (e.g., anorexia nervosa, muscle dysmorphia, compulsive exercise; [22]). Moreover, a lot of research on body image, and emotions related to it as well as physical activity, focuses on, e.g., [23,24]: (1) negative body image (there are few publications on positive body image), (2) the intensity of physical activity (without analysing in detail the motives for undertaking it). It is also crucial to mention that the intensity of a physical activity alters with age, which may also be associated with a change in the motives for engaging in it [25,26]. Therefore, in order to deepen the knowledge so far, it may be worth taking a closer look at the relationship between shame towards the body and motives for undertaking physical activity related to body image.

Importantly, younger women are characterized by a higher level of self-objectification than older women [27]. Therefore, in addition to the information presented above, in these analyses it seems important to take into account the age of the subjects, which, as can be concluded on the basis of previous studies, can be a differentiating factor for both body image and physical activity, e.g., [28,29,30,31]. While these studies often yield conflicting results, there are a number of them showing that over the years women begin to pay more attention to the health aspect of the body and its functionality rather than appearance itself [28,29,30] and this may result in the fact that a physical activity is practiced in many cases more to maintain health and the above-mentioned functionality than simply to lose weight and have a body in accordance with the current “beauty standards”.

Although knowledge about the relationship between self-objectification, physical activity and body image has increased in diverse populations [32], research and knowledge on body shame, motives for exercise, positive body image and the role of age in Polish women remains limited. Considering that body shame can lead to serious consequences such as eating disorders, depression and sexual dysfunction [33], it is vital to develop a fuller understanding of this issue in Polish women. Moreover, clarity on this issue is essential to ensure that an appropriate preventive activity and interventions are made which will be able to take into account the specific sociocultural context in Poland.

Therefore, the aim of this study has been to analyse whether body shame and age may play a role in appearance-based exercise and positive body image in women from Poland. It was assumed that women with high body shame and at the stage of young adulthood have significantly greater levels of appearance-based exercise and lower positive body image than those with a low level of body shame and at the stage of middle adulthood.

## 2. Materials and Methods

### 2.1. Participants and Procedure

This preliminary study was carried out from July to September 2022. All procedures were conducted in accordance with the Declaration of Helsinki and the study was approved by the Ethics Committee. All participants were informed about the purposes of the study, and participation was voluntary (without remuneration) and anonymous. Women were recruited via online advertising and snowball sampling. Participants interested in the study contacted the researcher and were included in the study (first step—consent to participate in the study; second step—completing the online survey).

The women came from the general population and were volunteers (a non-probability sampling technique). The two primary inclusion criteria were: (a) age over 18, (b) being female. Estimating the minimum sample size, the following recommendations were taken into consideration—“sample sizes of N = 20 to N = 30 per expected subgroup” [34] and “a sample size between 60 * k and 70 * k, where k equals the number of features” [35].

The final sample included 234 Polish women who ranged in age from 18 to 60 years (*M* = 31.58, *SD* = 13.93) and in self-reported body mass index (BMI) from 14.87 to 44.92 kg/m^2^ (*M* = 23.45, *SD* = 4.72). With regard to education, 2.56% (*N* = 6) completed primary, 45.30% (*N* = 106) secondary or technical school, and 52.14% (*N* = 122) had a higher education.

### 2.2. Measures

#### 2.2.1. The Objectified Body Consciousness Scale

Body shame was measured with the Objectified Body Consciousness Scale (OBCS), which has an adequate validity and reliability [36,37]. The Polish version of the OBCS covers 24 items making up 3 subscales related to objectification (“body shame”, “body surveillance”, “control beliefs”) to which participants respond on a 5-point scale (from “strongly disagree” to “strongly agree”). The higher the score, the higher the objectification measured by each subscale [27,28]. In this study, only one subscale was used—body shame—for which Cronbach’s alpha coefficient (reliability) was 0.89.

#### 2.2.2. The Exercise Appearance Motivations Scale

Appearance-based exercise was examined with the Exercise Appearance Motivations Scale (EAMS), which has an adequate validity and reliability [38]. The EAMS covers 32 items making up 5 subscales (“muscularity”, “appearance”, “societal pressures”, “shape/weight concerns”, “avoidance/shame”) to which participants respond on a 7-point scale (from “definitely disagree” to “definitely agree”). The higher the score, the greater the endorsement of appearance-based motives for exercise [38]. In this study, all subscales were used for which Cronbach’s alpha coefficients (reliability) were: (a) muscularity: 0.93, (b) appearance: 0.94, (c) societal pressures: 0.87, (d) shape/weight concerns: 0.96, (e) avoidance/shame: 0.94.

#### 2.2.3. The Body Appreciation Scale-2

Positive body image was assessed with the Body AppreciationScale-2 (BAS-2), which has an adequate validity and reliability [39,40]. The BAS-2 covers 10 items making up a total score related to body appreciation. In this measure participants respond on a 5-point scale (from “strongly disagree” to “strongly agree”). The higher the score, the higher positive body image (body appreciation) [39,40]. Cronbach’s alpha coefficient (reliability) was 0.96.

#### 2.2.4. Socio-Demographic Variables

Women also completed a socio-demographic survey (age, weight, height, educational qualification). Body mass index was calculated based on the self-reported weight and height [41]. With regard to age (using one of the more common theories, Erikson’s development), two age groups were distinguished: (a) young adulthood: from 18 to 40 years old, (b) middle adulthood: from 40 to 65 years old [42,43].

### 2.3. Statistical Analysis

IBM SPSS Statistic version 26 was used to carry out a two-step cluster analysis (with Schwarz’s Bayesian criterion) to identify clusters based on body shame and age (both variables are continuous) in women from Poland [44]. Not all assumptions for parametric methods have been met. Therefore, a Kruskal–Wallis test (with the Bonferroni adjusted *p*-value in the post hoc multiple comparisons) was used to evaluate differences between the clusters with regard to appearance-based exercise and positive body image (body appreciation). The significance level was defined as *p* < 0.05.

## 3. Results

### 3.1. Cluster Analysis of Body Shame and Age

The cluster analysis technique indicated four distinct clusters (Table 1): (a) Cluster 1 (*N* = 83): high body shame (*M* = 30.05) and young adulthood (*M*_age_ = 23.25); (b) Cluster 2 (*N* = 29): high body shame (*M* = 25.00) and middle adulthood (*M*_age_ = 51.76); (c) Cluster 3 (*N* = 88): low body shame (*M* = 15.70) and young adulthood (*M*_age_ = 23.74); (d) Cluster 4 (*N* = 34): low body shame (*M* = 11.15) and middle adulthood (*M*_age_ = 54.97).

Table 1 contains information on the comparisons of clusters in terms of BMI and educational qualification.

### 3.2. Comparison of the Four Clusters for Appearance-Based Exercise and Positive Body Image

The analysis with the usage of Kruskal–Wallis’ test indicated a significant effect of clusters on appearance-based exercise (all subscales of EAMS) and positive body image (total score of BAS-2—body appreciation). Table 2 presents results of multiple comparisons.

Referring to the most important findings, these results indicated that Cluster 1 (high body shame and young adulthood) had significantly greater levels of almost all exercise motives related to appearance (“muscularity”, “societal pressures”, “shape/weight concerns”, “avoidance/shame”) and a significantly lower level of positive body image than Cluster 4 (low body shame and middle adulthood). Interestingly, significant differences between these groups were not observed only in relation to the “appearance” subscale.

In relation to the rest of the outcomes, these significant differences were found with regard to “societal pressures”, “shape/weight concerns”, “avoidance/shame” and “body appreciation” between Cluster 1 and Cluster 3 (low body shame and young adulthood). In turn, for “appearance” the differences between Cluster 1 and Cluster 3 were at *p* = 0.073 and for “muscularity” were insignificant.

Moreover, Cluster 2 (high body shame and middle adulthood) scored significantly higher on “societal pressures” and “avoidance/shame”, and lower on “body appreciation” than Cluster 4 (for “shape/weight concerns” *p* = 0.055). Cluster 3 also had higher scores on the “muscularity” subscale and lower ones on body appreciation than Cluster 4.

In terms of other comparisons between clusters, no significant differences were obtained.

## 4. Discussion and Summary

The outcomes partially support the hypothesis, as higher levels of almost all subscales related to appearance-based exercise (“muscularity”, “societal pressures”, “shape/weight concerns”, “avoidance/shame”) and lower positive body image were observed in women with high body shame and at the stage of young adulthood compared with women with a low level of body shame and at the stage of middle adulthood. These results indicate that both body shame and age may contribute to the intensity of appearance-based exercise and positive body image deterioration. The results are consistent with some reports cited in the theoretical introduction, e.g., [6,18,19]. It may therefore mean that women in the younger age group feel greater shame about their body, which may be related to the fact that standards of appearance are more intensely internalized by this group, and social pressure to have the “perfect body” is more intense [45,46]. This is conducive to taking up behaviours that are aimed at changing those aspects of body that can nowadays be considered as determinants of attractiveness (e.g., muscularity, e.g., [45,47]). Moreover, it is the group (high body shame, young adulthood) that, due to strong pressure, may feel the greatest fear about their body and its inconsistency with the ideal, and undertake various types of behaviour to avoid criticism and negative assessment of appearance from other people [33,48]. All these behaviours (especially a tendency to avoidance behaviours, e.g., avoidance of the usage of tight clothing, situations related to meals, social meetings in which the body is exposed, looking at themselves in the mirror) will foster fear related to the body and decrease the positive attitude towards one’s own body [48,49]. In practice this means that a woman: (a) will not accept her body due to the fact that it does not conform to the standard of “beauty” in force, (b) will not take into account the fact that every human body is unique and exceptional, and external appearance is also influenced by genetic factors, (c) will not feel comfortable in her body and will feel such emotions as, among others, sadness, fear and guilt in connection with it, (d) will focus excessively on her own body’s faults and will not appreciate its benefits, (e) will become convinced that the basic determinant of a person’s value is their external appearance. However, in order to verify exactly whether this mechanism works in this way (described above), further experimental or longitudinal studies should be carried out in this area, for which the results of this preliminary study may be indicative.

Moreover, some of the remaining (additional) comparisons between clusters confirm the assumption about the importance of shame for engaging in maladaptive behaviours, as evidenced by the differences between clusters 2 and 4, as well as 1 and 3. These results indicate that, if we compare groups that are identical in terms of age but differ in the level of shame, people experiencing a high level of body shame are characterized by less correct functioning in the selected subscales of EAMS and the BAS-2 scale. It may mean that, regardless of age, shame towards the body may foster problematic behaviours towards the body and consolidation of negative beliefs about it. Importantly, drawing attention to the fact that the aspect described in the theory of objectification—body shame—is so important may be crucial because, as it is well known, the mechanisms described above significantly increase the risk of the emergence of, among other things, eating disorders, muscle dysmorphia, performing plastic surgeries harmful to health, depression and compulsive exercise [24,33,50,51,52].

When analysing the context related to practising physical activity, it should be remembered that whether physical activity is pro-health also depends on the underlying motivation [44,45,46,47,48]. If physical exercise is practiced solely for the purpose of altering appearance and/or body/weight control, it may favour the development of compulsive exercise, which is known to have numerous negative consequences for physical and mental health (including eating disorders) and social functioning [53,54,55,56,57].

These studies were only preliminary studies and have certain limitations. The main ones are [29,58]: (a) small sample size, (b) age and educational differences (which may be a factor that may distort the results), (c) voluntary sampling, (d) cross-section studies (not able to infer cause and effect), (e) subjective measures, (f) lack of in-depth analysis of other types of motivation to exercise (e.g., due to health, to regulate emotions; e.g., The Exercise Motivations Inventory-2). Due to the above-mentioned information and the fact that, although the measures used have good reliability and validity, not all of them have been previously validated in a representative Polish sample, the obtained results should be approached with caution (because the outcomes may have a significant bias). Therefore, it is necessary to conduct further analyses in order to verify the previous reports and obtain an even better and clearer picture in terms of variables related to the objectification theory, physical activity, body image and age. Importantly, when undertaking further research attempts, one should especially take into account age differences and conditions typical for age, and consider designing a survey together with an objective measurement (e.g., body composition).

Undertaking further research in the scope described above is also crucial as, so far, many analyses have focused mainly on younger people, and knowledge of the analysed issues (i.e., body shame, motivation to exercise, positive body image) and the relationship between them in older women is limited. Therefore, it makes it difficult to prepare appropriate prevention and intervention programs, which would take into account, inter alia, the “age” factor. Knowledge in this area may prove helpful in building pro-health motives for practising a physical activity and developing skills in the field of adaptative behaviours related to taking care of your own body. It may also be important for the reason that it will favour the acceptance of the aging process and related body changes in the older age group, and thus protect people against deterioration of quality of life [29,59,60]. Moreover, the prevalence of the above-mentioned disorders (i.e., eating disorders) has increased recently in older women, and knowledge of the factors contributing to this phenomenon in this group is still very limited, e.g., [61]. Therefore, knowing that shame related to the body can also be significant in older women, it is worth exploring the operation of these mechanisms.

## Figures and Tables

**Table 1:** Body mass index and education level in the four clusters.

	CLUSTER 1 (*N* = 83): High Body Shame + Young Adulthood	CLUSTER 2 (*N* = 29): High Body Shame + Middle Adulthood	CLUSTER 3 (*N* = 88): Low Body Shame + Young Adulthood	CLUSTER 4 (*N* = 34): Low Body Shame + Middle Adulthood	
	***M* (*SD*)**				***Post hoc***
	*H*(3) = 43.14, *p* < 0.001				
**BMI**	24.18 (5.57)	26.60 (4.11)	21.41 (3.49)	24.28 (3.47)	1 vs. 2 ** 1 vs. 3 *** 1 vs. 4 2 vs. 3 *** 2 vs. 4 3 vs. 4 **
	***N* (%)**				***χ*^2^ and Cramer’s *V***
**Educational qualification** Primary school Secondary or technical school Higher education					*χ*^2^_(12)_ = 53.49 Cramer’s *V* = 0.28 ***
	4 (%)	1 (%)	1 (%)	0 (%)	
	49 (%)	4 (%)	49 (%)	4 (%)	
	30 (%)	24 (%)	38 (%)	30 (%)	

** *p* < 0.01; *** *p* < 0.001.

**Table 2:** Post hoc tests.

	CLUSTER 1 (*N* = 83): High Body Shame + Young Adulthood	CLUSTER 2 (*N* = 29): High Body Shame + Middle Adulthood	CLUSTER 3 (*N* = 88): Low Body Shame + Young Adulthood	CLUSTER 4 (*N* = 34): Low Body Shame + Middle Adulthood	
	***M* (*SD*)**				***Post hoc***
**Appearance-based exercise (EAMS)**					
	*H*(3) = 17.28, *p* < 0.001				
Muscularity	6.05 (3.35)	4.55 (3.26)	6.07 (4.03)	3.74 (2.93)	1 vs. 2 1 vs. 3 1 vs. 4 *** 2 vs. 3 2 vs. 4 3 vs. 4 **
	*H*(3) = 8.70, *p* < 0.05				
Appearance	44.80 (11.24)	41.52 (13.02)	41.28 (11.77)	42.06 (9.73)	1 vs. 2 1 vs. 3 ^†^ 1 vs. 4 2 vs. 3 2 vs. 4 3 vs. 4
	*H*(3) = 40.08, *p* < 0.001				
Societal pressures	35.67 (10.32)	31.28 (10.20)	27.85 (10.45)	23.59(6.54)	1 vs. 2 1 vs. 3 *** 1 vs. 4 *** 2 vs. 3 2 vs. 4 * 3 vs. 4
	*H*(3) = 42.53, *p* < 0.001				
Shape/weight concerns	37.25 (11.69)	32.72 (11.53)	25.66 (14.11)	23.71 (9.65)	1 vs. 2 1 vs. 3 *** 1 vs. 4 *** 2 vs. 3 2 vs. 4 ^††^ 3 vs. 4
	*H*(3) = 56.68, *p* < 0.001				
Avoidance/shame	25.60 (8.31)	22.62 (7.16)	17.41 (9.20)	12.68 (7.43)	1 vs. 2 1 vs. 3 *** 1 vs. 4 *** 2 vs. 3 2 vs. 4 ** 3 vs. 4
**Positive body image (BAS-2)**					
	*H*(3) = 77.06, *p* < 0.001				
Body appreciation	2.84 (0.88)	3.24 (0.73)	3.66 (0.92)	4.51 (0.49)	1 vs. 2 1 vs. 3 *** 1 vs. 4 *** 2 vs. 3 2 vs. 4 *** 3 vs. 4 ***

EAMS—the Exercise Appearance Motivations Scale; BAS-2—the Body Appreciation Scale-2; * *p* < 0.05; ** *p* < 0.01; *** *p* < 0.001, ^†^ *p* = 0.073, ^††^ *p* = 0.055.

## Data Availability

The data that support the findings of this study are available from the corresponding author upon reasonable request.
